# Joint Interaction and Mutual Understanding in Social Robotics

**DOI:** 10.1007/s11948-022-00407-z

**Published:** 2022-10-26

**Authors:** Sebastian Schleidgen, Orsolya Friedrich

**Affiliations:** FernUniversität in Hagen, Institute of Philosophy, Universitätsstrasse 33, 58097 Hagen, Germany

**Keywords:** Anthropomorphism, Human–Machine interaction, Human–Robot communication, Cybernetics, Phenomenology, Social cognition

## Abstract

Social robotics aims at designing robots capable of joint interaction with humans. On a conceptual level, sufficient mutual understanding is usually said to be a necessary condition for joint interaction. Against this background, the following questions remain open: in which sense is it legitimate to speak of human–robot joint interaction? What exactly does it mean to speak of humans and robots sufficiently understanding each other to account for human–robot joint interaction? Is such joint interaction effectively possible by reference, e.g., to the mere ascription or simulation of understanding? To answer these questions, we first discuss technical approaches which aim at the implementation of certain aspects of human–human communication and interaction in social robots in order to make robots accessible and understandable to humans and, hence, human–robot joint interaction possible. Second, we examine the human tendency to anthropomorphize in this context, with a view to human understanding of and joint interaction with social robots. Third, we analyze the most prominent concepts of mutual understanding and their implications for human–robot joint interaction. We conclude that it is—at least for the time being—not legitimate to speak of human–robot joint interaction, which has relevant implications both morally and ethically.

## Introduction

This paper is dedicated to the question of whether, or rather: in what sense it is legitimate to speak of human–robot joint interaction. In what sense can we speak of humans and robots sufficiently understanding each other to account for human–robot joint interaction? Alternatively, could such joint interaction also be effectively possible by reference, e.g., to the mere ascription or simulation of understanding? To answer these questions, we, first, provide some general background on modern social robotics. Second, we go into some detail regarding the technical approaches to implement certain aspects of human–human communication and interaction in social robots with the aim of making robots accessible and understandable to humans and, therefore, human–robot joint interaction possible. Third, we examine the human tendency to anthropomorphize in this context with a view to human understanding of and joint interaction with social robots. Fourth, we analyze the most prominent concepts of mutual understanding and their implications for human–robot joint interaction. This provides the backdrop against which we, fifth, provide an answer to the question in what sense we may speak of human–robot joint interaction. As will be shown, all approaches analyzed are either implausibly reductionist or link demands to mutual understanding that make it questionable whether artificial entities like social robots can satisfy them appropriately. As a consequence, it is, at least for the time being, not legitimate to speak of human–robot communication, understanding, and joint interaction.

## Background

Modern social robots are expected to interact with humans in various contexts and in ways that are accessible and understandable to humans. Accordingly, social robotics aims at developing “[…] autonomous or semiautonomous robots that are natural and intuitive for the general public to interact with, communicate with, work with as partners, and teach new capabilities” (Breazeal et al., 2016; cf. Pipitone & Chella, 2021 for a recent example). Usually, three types of social robots are distinguished (Sandry, 2015a, 2015b): *human-like* or *android robots*, *animal-like* as well as *machine-like* or “overtly other” robots. Social robots are used in different contexts and for different purposes (Table 1), and make use of different communication modalities to interact with humans, such as whole-body motion, proxemics, gestures, facial expressions, gaze behavior, head orientation, linguistic or emotive vocalization (Breazeal et al., 2016).

**Table 1:** Exemplary purposes of social robots

Robotic purpose	Examples
Verbal communication	Mel (Sidner et al., 2005)
Nonverbal communication	Pepper (Wada et al., 2006) Kismet (Jokinen, 2018)
Emotional communication	AIBO (Fujita, 2004) Keepon (Kozima, 2006) ROMAN (Berns & Hirth, 2006) WABIAN (Ogura et al., 2006) WE-4RII, WF-4RII (Itoh et al., 2006; Solis et al., 2006)
Communication-based services	Valerie (Gockley et al., 2006)
Support of people with communication problems	KASPAR (Dautenhahn et al., 2009)
Shared attention and collaboration	Leonardo (Breazeal et al., 2004)
Education	NAO (Pollack et al., 2002)
Elderly Care	Paro (Wada et al., 2006) Pearl (Jokinen, 2018)
Study effects of human presence	Geminoid (Shimada et al., 2006)
Study social signaling	Nexi and Maddox (Jung et al., 2013)

From the very beginning, social robotics aimed at developing robots not only entertaining interaction with the purpose of being accessible and understandable, but being capable of *joint action* with humans (Clodic & Alami, 2021; Clodic et al., 2017; Glasauer et al., 2010; Grigore et al., 2013; Jokinen, 2018; Mörtl et al., 2014). Joint action is generally understood as a specific form of interaction with “two or more individuals coordinat[ing] their actions in space and time to bring about a change in the environment” (Sebanz et al., 2006).^1^ Human–robot joint action, hence, essentially refers to coordinated and goal-directed action of at least two (human and robotic) individuals.

This raises the question of the conceptual presuppositions as well as the requirements of human–robot joint action. In social robotics, there are two main approaches to solve the problem of enabling robotic joint action with humans: on the one hand, attempts that refer to *human–human* joint action and aim at implementing certain human characteristics in robots deemed relevant for joint action (Breazeal et al., 2016). On the other hand, accounts assuming a general impossibility of such implementation and, hence, calling for an alternative understanding of *human–robot* joint action and opposing efforts to implement human characteristics in robots for the purpose of joint action (Peters, 1999; Sandry, 2015b). Social robotics mainstream mostly follows the first approach (Breazeal et al., 2016). This, however, shifts the question of the requirements of human–robot joint action towards the question of the requirements of *human–human* joint action as well as to possibilities of their implementation in robots.

*Human–human* joint action is usually said to at least depend “on the abilities (i) to share representations, (ii) to predict actions, and (iii) to integrate predicted effects of own and others’ actions” (Sebanz et al., 2006; cf. Gallagher, 2020). Although this has provoked extensive debates on the exact determination of the necessary conditions for joint action, particularly on the role and meaning of “shared intentions”, “joint attention”, and “task sharing”, as well as “action observation” and “coordination”, it is widely undisputed that human–human joint action requires sufficient *mutual understanding* of the involved individuals, at least with a view to the relevant aspects regarding the context and purpose of a joint action (Bratman, 1993, 2013; Breazeal et al., 2016; Butterfill, 2012; Clodic & Alami, 2021; Gallagher, 2020; Tomasello et al., 2005). Such understanding may refer to, among other things, the emotions, feelings, reasons, and intentions of others. In general, this means an understanding of certain mental states of others, to make their actions sufficiently predictable. Ultimately, only the satisfaction of this very condition of sufficient mutual understanding makes the integration of predicted action effects—and, hence, joint action as goal-directed coordinated action—possible at all (Overgaard & Michael, 2015; Robillard, 2006; Strasser, 2020). It is crucial that sufficient *mutual* understanding generally refers to, or is based on, *interactions* of different kinds and on different levels between at least two entities. Therefore, we prefer the term of *joint interaction* over the term of joint action when it comes to mutual understanding.

It remains unclear, however, what consequences this reference to mutual understanding has for joint interaction of humans and robots, especially if one orients human–robot joint interaction to human–human joint interaction. A preliminary answer may be: if joint interaction requires sufficient mutual understanding of the involved parties, then sufficient mutual understanding of humans and robots (and, hence, the satisfaction of its conditions) is necessary for successful human–robot joint interaction. It follows that human agents would have to sufficiently understand their robot partners in contexts of joint interaction *and *vice versa (Clodic & Alami, 2021). In this context, human-like and animal-like robots have proven to be particularly promising (Breazeal et al., 2016; Sandry, 2015b). This is due to the strong tendency in humans to *anthropomorphize* or *zoomorphize* robots, i.e., to ascribe “for instance emotional states, motivations, intentions […] *by the user* to the robot” (Lemaignan et al., 2014; cf. Epley et al., 2008) resulting in humans being convinced of *understanding* robots (e.g. Banks, 2020; Fink, 2012; Reeves & Nass, 1996; Schreck et al., 2019). This ultimately led to the focus on anthropomorphic (and zoomorphic) design principles (Kiesler et al., 2008; Lemaignan et al., 2014; Powers et al., 2003), as well as to approaches to implement the above-mentioned communication modalities in social robots.^2^ The question arises, however, whether this follows a plausible conceptualization and may result in a corresponding realization of sufficient human understanding with a view to human–robot joint interaction.

As regards sufficient *robotic* understanding of human agents in the context of joint interaction, however, the matter is more complicated. It seems obvious, as Strasser (2020) puts it, that “if artificial agents qualify as social agents in a joint action, we have to expect [the ability to understand others] from them”. In view of this, numerous concepts of and approaches to an artificial theory of mind (Bianco & Ognibene, 2019; Butterfill & Apperly, 2013; Chen et al., 2021; Duarte et al., 2018; Iwahashi, 2003; Scassellati, 2002; Strasser, 2020; Vanderelst & Winfield, 2018; Winfield, 2018), or, more broadly, of robotic understanding (Clodic et al., 2017; Hellström & Bensch, 2018; Jacq et al., 2018) have been developed in contexts of human–robot joint interaction. However, these approaches mostly focus exclusively on the ability of social robots to statistically predict human intentions and actions (Sciutti et al., 2018). In many contexts, this approach seems to work well and human–robot joint interaction to be successful. In analogy to the case of humans (supposedly) understanding robots, however, the question arises whether this implies a plausible conceptualization and may result in a corresponding realization of sufficient robotic understanding with a view to human–robot joint interaction. Authors like Fiebich et al. (2015) seem to be affirmative although not explicitly concerned with the issue of robotic understanding.

Others dispute the key premise of Strasser, namely that any agent to qualify as a social agent in a joint interaction must have certain capacities, e.g. the ability to understand others. According to them, this, in fact, would imply the impossibility to treat robots as social agents and, hence, to legitimately speak of human–robot joint interaction. Instead, they postulate “new conceptual tools for forms of non-reciprocal or asymmetric sociality, i.e., for social interactions where one agent lacks the capacities required for normative agency” (Seibt, 2017). This leads to the proposal that robots may simulate (joint) interaction in different ways, resulting in different degrees of sociality and, with that, joint interaction (Seibt, 2014, 2017). If this holds true, at least certain robots would indeed qualify as social agents (to some degree), and certain human–robot interactions would qualify as joint interactions (to some degree).

The question of how to conceptualize and eventually realize robotic understanding with a view to human–robot joint interaction is not merely conceptually interesting, but of key relevance for normative considerations. On the one hand, it is *morally* relevant in the sense that any answer to it influences our everyday moral beliefs, rules, norms and values. For instance, the way we see and talk about robots, e.g., as joint interactors, significantly influences our perceptions and moral expectations of robots (within the boundaries of our moral community). On the other hand, answering the question of robotic understanding is *ethically* relevant, insofar it is vital for a (philosophical or scientific) reflection on moral phenomena regarding the relationship of humans and robots. For instance, the attribution of certain properties and abilities to robots may imply certain rights of or duties towards robots. Ultimately, speaking of human–robot joint interaction implies that we are dealing with robotic social *agents* whom we should treat in a certain way or from whom we would expect to be treated in a certain way, who have certain responsibilities in joint interactions or are responsible for certain consequences of joint interactions. In other words: the question of whether it is legitimate to speak of human–robot joint interaction may be relevant for the question of whether it is ethically justified to treat robots as moral subjects or moral patients (Damiano & Dumouchel, 2018; Nye & Yolbas, 2021; Nyholm, 2020; Véliz, 2021). In the light of these normative dimensions, non-reciprocal or asymmetric sociality, as postulated, e.g., by Seibt (2014, 2017) seems implausible as it would either result in lowering established moral or ethical standards in view of the capabilities of robots or lead to double-standards with a view to the concept of joint interaction.

Having laid out this, we may also, in contrast to, e.g., Hakli (2014), *not* “argue that if people conceive of their interaction with robots as social interaction, this should count as prima facie evidence that their interaction with robots is social interaction “. Instead, from an ethical perspective, we must conceptually analyze any claim of human–robot social, e.g., joint interaction, at least with a view to its potential moral and ethical implications. For only such analysis can provide an appropriate foundation for deciding whether robots may count as social agents and joint interactors.

## The Technology Behind “Understanding” and “Understandable” Social Robots

When it comes to promoting the accessibility as well as understandability of robots and, hence, human–robot joint interaction, the mainstream of social robotics focuses on the implementation of certain human, or at least: human-like, characteristics and abilities in robots (Brinck & Balkenius, 2020). With a view to the understandability of robots, *communicative* characteristics and abilities are deemed particularly vital. In this regard, two main issues arise: first, the question of an appropriate robotic *communication repertoire*, i.e., the set of abilities considered necessary for adequate communication with humans. And second, the issue of implementing corresponding *communication architectures*, i.e., the concrete technical realization of these very abilities in robots. As regards the communication repertoire, social-emotional intelligence as well as socio-cognitive skills are at the center of considerations (Breazeal et al., 2016; cf. Clodic et al., 2017; Goswami & Vadakkepat, 2019; Nyholm, 2020; Von Braun et al., 2021): depending on the intended use and context, it is deemed necessary that robots are able to recognize and interpret affective signals from humans, that they possess internal models of emotions (usually based on certain psychological theories of emotion), and that they are able to communicate affective states. Furthermore, to allow for successful human–robot joint interaction, social robots should be able to recognize, understand, and predict human behavior with a view to its underlying mental states (such as beliefs, intents, desires, and feelings) and react accordingly.

As regards the technical realization of these abilities, social robotics strongly focusses on possible ways to—verbally and non-verbally—exchange information (cf., e.g., Bisk et al., 2016; Edwards et al., 2020). For verbal exchange, *natural language processing* (NLP) modules are implemented to communicate *primary* information either in turn-based or in dynamic conversations that may account for social signals and other background information relevant for human–robot dialogue interactions in a dynamically changing world (cf. Jokinen, 2018). As regards non-verbal information-exchange, different types of regulators for gaze and pose behavior, displays indicating affective, cognitive, or conversational states, illustrators allowing for deictic and iconic gestures, as well as sensors and mechanisms for tactile interaction are used to communicate *secondary* information intended to supplement verbally exchanged information (Breazeal et al., 2016).

Besides allowing robots to understand humans, the overarching goal of these efforts consists in making robots understandable to humans and, hence, in increasing human willingness to engage with robots, to interact cooperatively and effectively, and, ultimately, to jointly interact with them (Breazeal et al., 2016; Sandry, 2015b). In this regard, *anthropomorphic design principles* play an important role, which refer to the much-discussed human tendency to *anthropomorphize* non-human beings and entities. This, however, requires elaborating what the concept of anthropomorphism and the tendency to anthropomorphize alludes to. In particular, such an elaboration is necessary concerning the question of sufficient mutual understanding as a necessary condition for legitimately speaking of human–robot joint interaction.

## Questioning the Significance of Anthropomorphizing Social Robots for Mutual Understanding and Joint Interaction

Generally, anthropomorphism is understood as “the human tendency to attribute human traits to non-human entities” (Damiano & Dumouchel, 2018; cf. Epley et al., 2007; Złotowski et al., 2015), or to interpretate “non-human behavior as motivated by human feelings and mental states” (Airenti, 2015). Hence, the concept does not only refer to the human-like *appearance* of non-human beings or entities, but also to the *attribution* of certain human *competencies* and *characteristics*.

As regards appearance, the tendency to anthropomorphize has, for instance, been demonstrated for robots with humanlike faces and voices (Nass & Brave, 2005; Yee et al., 2007), or other bodily and behavioral *similarities* or *commonalities* (Ames, 2004). As regards the attribution of competencies and characteristics, in view of certain robotic features people tend to assume that robots have, e.g., certain levels of knowledge regarding specific facts and phenomena, that they have certain characters, or attitudes towards humans, objects, as well as social phenomena (Lee et al., 2005; Powers et al., 2005). This often implies projecting one’s attitudes onto robots as well as developing specific attitudes towards robots, sometimes culminating in cooperating with robots “as […] with a real person” (Kiesler et al., 2008). Therefore, while certain similarities or commonalities of humans and robots seem to be a necessary condition for anthropomorphizing robots, ongoing human–robot interactions may even *increase* the degree to which humans perceive robots as being similar or sharing commonalities and, hence, the degree of accepting robots as cooperators (Sandry, 2015a).

The projecting of one’s attitudes is of particular interest in this context, as it shows the crucial role of pre-existing attitudes for the ways in which robots are anthropomorphized, as well as for the degree of human willingness to cooperation and joint interaction: this willingness decisively depends on the basic attitudes and values one holds, especially when it comes to interaction with non-human beings and entities (Richert et al., 2018). This is what Kiesler et al. (2008) have in mind when stating that “anthropomorphism is partly a value prescription process that facilitates potential interaction” and, ultimately, communication and joint interaction. This is one of the reference points for understanding “anthropomorphism as a fundamental tool in successful human–robot relations” (Damiano & Dumouchel, 2018) and, accordingly, for introducing anthropomorphic design principles in social robotics. For “then face-to-face interaction with a humanlike machine should prompt greater anthropomorphism of the machine” (Kiesler et al., 2008) and, in turn, support further interaction, communication, and joint interaction. According to this position, the tendency to anthropomorphize is not understood as a cognitive mistake, as is often argued, but rather as a basic tool that can be used to improve human–robot interaction: “humanlike […] social robot designs are based on the assumption that familiar appearance and modes of communication […] are the best way to support easy and effective human–robot interactions” (Sandry, 2015a). However, this has not remained uncriticized. From an ethical perspective, for instance, a number of authors is concerned that anthropomorphizing robots could result in cognitive and psychological damage or even in reduced quality of life (Damiano & Dumouchel, 2018; cf. Allen & Wallach, 2012; Calo, 2012; Whitby, 2012).

More important for our purpose, however, are critiques that focus on the connection of anthropomorphizing robots on the one hand and understanding them on the other: in this regard, one line of fundamental criticism is of particular interest. Following the Levinasian tradition, “the other always retains a level of alterity and therefore cannot be completely comprehended by the self” (Sandry, 2015a). Communication with and understanding the Other, according to this position, necessarily points toward, or even is reliant on, the self. Consequently, binding communication, understanding and joint interaction to certain commonalities with humans—as anthropomorphic design principles do—would only result in “[c]ommunication as reduplication of the self or its thoughts in the Other” (Peters, 1999). While, according to this position, the persisting difference between the self and the Other would make communication necessary in the first place, creating merely external similarities between the self and the Other *per se* does not allow for communication or even mutual understanding, but rather only for projecting one’s own attitudes, beliefs and intentions in the Other (here: the robot) (Sandry, 2015a).

A second line of argument holds that making use of the human tendency to anthropomorphize robots could be deceptive and, hence, lead to false expectations. According to this argument, the anthropomorphic design of robots would present them to humans as “artifacts that have inner states of mind”. Therefore, human–robot interaction could promote the *illusion* of “understanding these states of mind” (Turkle, 2005; cf. Damiano & Dumouchel, 2018) and, as a consequence, maybe even expectations of robots being capable of understanding humans or following human social norms and reciprocity conditions (Syrdal et al., 2008; cf. Złotowski et al., 2015). This is not necessarily to say that the human tendency to anthropomorphize is, in principle, a cognitive mistake (even though Turkle seems to take this position), but rather that it is misleading when exploited in designing robots and, as such, having many important implications for, e.g., human self-understanding.

In summary, it seems to be true that an anthropomorphic design of robots affords the *attribution* of certain traits, abilities, and characteristics (e.g., knowledge, intelligence, understanding) (Lee et al., 2005; Powers et al., 2005) and, hence, fosters human *willingness* to engage with robots, to interact cooperatively and effectively, and, ultimately, to jointly interact with them (Bailenson & Yee, 2005; Kiesler et al., 2008; Parise et al., 1999; Torrey et al., 2006). At the same time, however, this attribution and willingness do only seem to establish a certain kind of *one-way relation*: attributing certain traits, characteristics and abilities to robots ultimately is the result of *projecting* human values, attitudes, characteristics, and abilities onto robots, which, in turn, promotes willingness to interact with a view to certain human goals (for a more detailed discussion of this point cf., e.g., Brinck & Balkenius, 2020; Damiano & Dumouchel, 2018; Duffy, 2003).

At this point, recall the necessary conditions for joint interaction: it requires successful communication with the goal of sufficient *mutual understanding*, at least with a view to the relevant aspects regarding the context and purpose of a joint interaction. In this regard, successful communication may presuppose, among other things, sufficient understanding of the emotions, feelings, reasons, and intentions, in general: of the mental states of others, to make their actions sufficiently predictable with a view to goal-directed coordinated action. Therefore, it remains questionable whether mere attribution or projection (e.g., of robots communicating with and understanding humans) is sufficient to appropriately speak of humans understanding robots. Vice versa, it is also questionable whether existing robotic communication repertoires and architectures (such as the ones outlined above) are sufficient to appropriately speak of robots understanding humans. Hence, the question remains whether, and if so, in what sense, an anthropomorphic design of robotic communicative characteristics and abilities allows for sufficient human–robot mutual understanding as a necessary condition for joint interaction. Ultimately, this alludes to the question of whether mere attribution of certain characteristics and abilities as communicative is sufficient, or whether these properties must in fact be realized in robots to foster (mutual) understanding and possibly joint interaction. To answer this question, it is worth taking a more detailed look at the most important approaches and theories dealing with communication and (mutual) understanding.

## Communication and Mutual Understanding

Craig (1999) identified seven traditions of communication theory: first, *rhetorical* approaches, which theorize communication as “the practical art of discourse”; second, *semiotic* approaches defining communication as “intersubjective mediation of signs”; third, *phenomenological* approaches which grasp communication as “experience of otherness” and dialogue; fourth, *cybernetic* approaches conceptualizing communication as “information processing”; fifth, *sociopsychological* approaches, which capture communication as “expression, interaction & influence”; sixth, *sociocultural* approaches conceiving communication as “(re)production of social order”; and seventh, *critical* approaches framing communication in terms of “discursive reflection”. Such different approaches to communication result in highly differing conceptions of understanding. Although almost all these approaches share certain aspects and can in principle be used to analyze and evaluate different aspects of social robots and human–robot interaction, the cybernetic and the phenomenological accounts seem particularly interesting concerning our question of (mutual) understanding and joint interaction with robots.

### Communication and Mutual Understanding in the Cybernetic Tradition

It is often claimed that the cybernetic tradition initiated modern communication theory, following the work of, e.g., Claude E. Shannon, Warren Weaver, John von Neumann, and Alan Turing (Craig, 1999; Heims, 1991; Krippendorff, 1989). Simultaneously, the influence of cybernetics is still observable in current theories in, e.g., systems and information science, cognitive science or artificial intelligence (Craig, 1999). Thus, it is still an important point of reference in information technology and social robotics development. Cybernetics theorizes communication as *mathematically quantifiable and predictable information processing and exchange,* which can take place in and between all kinds of complex systems. Accordingly, not only human individuals may communicate with each other, but also humans and artificial entities (like, e.g., robots), groups, organizations, and whole societies. Furthermore, individual thought of living and non-living entities is framed as intrapersonal communication, resulting from the idea that thought would be “nothing more than information processing” (Craig, 1999).

As a consequence, successful communication in and between entities “is linked with the clear transmission or exchange of accurate information” in this model (Sandry, 2015a). Semantic aspects of information, however, are excluded from this model (Shannon, 1948). This is due to the aim of Shannon (and other proponents of the transmission model) to mathematically quantify and predict information and communication: as meaning depends on semantic interpretation of information, which, in turn, is essentially dependent on subjective preconditions of individuals involved in communication, it would seem not appropriate to quantify and predict it mathematically and, hence, to include it in the transmission model (Kimmel, 2020).^3^

Concerning our question of (mutual) understanding and human–robot joint interaction, it could be stated that, according to the transmission model, successful communication of some information from a robot to a human (or vice versa) would result in some sense of understanding in this very human (or robot). However, such successful communication is solely measured in terms of the mathematical predictability for information being appropriately reproduced in the receiver: “[t]he fundamental problem of communication is that of reproducing at one point either exactly or approximately a message selected at another point” (Shannon, 1948). Even mental states would then have to be regarded as or as a result of information processing, and would have to be grasped and transferred mathematically accurate with a view to communication and understanding. This is probably the reason why cybernetics and the transmission model continue to play an important role in machine learning (ML), robotics and particularly in the development of anthropomorphic robots (Sandry, 2015b; cf., e.g., Li et al., 2016 for a mathematical model of conversational agents). We doubt, however, that this model is appropriate to make the actions of joint interactors sufficiently predictable with a view to goal-directed coordinate action: its inherent reduction of communication to mathematically quantifiable and predictable information processing and exchange does not seem to account for any plausible version of human communication, understanding and joint interaction.

### Communication and Mutual Understanding in Phenomenology^4^

A rather different account of communication and (mutual) understanding follows from the phenomenological tradition. Interestingly, its founder Edmund Husserl (2012 [1931]) emphasized the importance of commonalities between individuals involved in communicative processes. In view of this, and similar to the cybernetic tradition, his theory is sometimes said to be “the very foundation of modern theories of communication” (Chang, 1996) as well as to be reflected in the assumption of modern social robotics “that humanlike robots are likely to make more effective social robots” (Sandry, 2015a). In fact, there seems to be some similarity between Husserl’s approach and the cybernetic tradition, although Husserl did not allude to a mathematical reduction of communication, but rather to commonalities of form and behavior in communicating individuals.

In contrast, phenomenologists like Emmanuel Levinas focus on the value of difference between individuals, which ultimately would make it impossible to completely understand the Other. As a consequence, accounts of communication presupposing similarities or commonalities of individuals in terms of successful communication and understanding would fall prey to inauthenticity (Levinas, 1989). This is the reason why phenomenologists of this tradition often criticize social robotics and particularly the development of anthropomorphic robots. Indeed, authors like Pinchevski (2005) evaluate traditional communication theories as, for instance, those of cybernetics, to be violent because their “descriptions of communication can be understood to support the elimination of otherness and difference, resulting in a level of ‘violence’ to the other” (Sandry, 2015b).

It is important, however, that, according to Levinas, this difference between individuals is the very reason for the necessity of communication in the first place (Levinas, 1969, 1989; cf. Sandry, 2015a). As regards our question of (mutual) understanding as necessary condition for human–robot joint interaction, according to this concept of communication, understanding the other and, hence, mutual understanding is principally problematic. With, e.g., Stegmaier (2020), the strongest possible claim seems to be that understanding the other consists of our “permanent testing of interpretations we expect others may have”. The implications of this for human–robot joint interaction remain, however, open. However, it could also be followed that robotic communication and understanding are not readily comparable to human–human communication and understanding and, hence, that a completely different approach is needed for human–robot joint interaction (Damiano & Dumouchel, 2018; Duffy, 2003; Stegmaier, 2020; Złotowski et al., 2015). It still remains an open question how such an approach could be conceptualized. Such a speculative position therefore does not allow for an answer to our question regarding human–robot joint interaction.

### Communication and Mutual Understanding in Social Cognition Theories

Regarding the abilities of understanding the mental states of others as well as anticipating or predicting their behavior (possibly with the aim of goal-directed coordinate and, hence, joint interaction), a third line of theories comes into play, which relates these abilities to so-called “mindreading” or “Theory-of-Mind” (Fodor, 1992). Roughly, theories of this kind can be divided into *cognitivist* and *interactionist* theories (De Jaegher et al., 2010; Gallotti & Frith, 2013). The most important variants of the cognitivist theory are the well-known approaches of *theory-theory* and *simulation theory*.

*Theory-theory* (as developed, e.g., by Gopnik & Wellman, 1992; Leslie, 1994) assumes that mindreading and, hence, the ability to understand others’ mental states, requires a Theory-of-Mind. Accordingly, mindreading is based on implicit knowledge of a set of “folk” laws or principles that link mental states to sensory stimuli, behavioral responses, and other mental states (Barlassina & Gordon, 2017). Hence, the ability to represent and think about the mental states of others is said to be based on a tacit psychological theory, and mindreading is defined as an information-rich and a theory-driven process. However, approaches of theory-theory differ as to where this information stems from. According to Overgaard and Michael (2015), it is either.

- [a] “theory” that is formed on the basis of observation, testing, and learning […]; or:
- [c]ontained in a “module” that is activated at some point in development […].

In contrast, proponents of *simulation theory* (e.g., Gordon, 1986; Heal, 1995) hold mindreading to be a matter of taking others’ perspectives, where mental simulation plays a central role. Hence, when we mindread, we represent the mental states of others by simulating their mental states in our own minds, “by putting ourselves in other people’s ‘shoes,’ using our own mind to work out what we would do, think, or feel in their situation—and then attributing those intentions, thoughts, or emotions to those other people” (Overgaard & Michael, 2015). However, if this holds true, simulation theorists continue, i.e., if our cognitive resources can be modified to function as representations of other people’s mental states, then we do not need to store general information about how people function (Barlassina & Gordon, 2017). Accordingly, mindreading is defined as information-poor and purely process-driven.

As regards our question of (mutual) understanding as necessary condition for human–robot joint interaction, it becomes clear that referring to either theory-theory or simulation theory would set fairly high standards for the development of social robots. First, “a wide range of complex mental states” (Strasser, 2020) would be required, including, e.g., desires and emotions. Second, a psychological theory (in a theory-theory-driven approach) —either acquired through learning or implemented in some module –, or the ability to simulate mental states of others (in a simulation-theory-driven account) would be necessary. In order to qualify as mindreaders, robots (and humans) would not only “have to notice that another agent is noticing something relevant for the joint action, but […] also [have to] recognize whether the other agent is desiring something or is afraid of something” (Strasser, 2020). Although, in fact, much research is done on these subjects, they still are a particularly challenging aspect in the design and development of social robots.

A solution to this problem could be to elaborate certain minimal conditions of mindreading and, consequently, of (mutual) understanding and joint interaction, as, for instance Butterfill and Apperly (2013) have presented in their account of a *Minimal Theory of Mind*. The authors propose tying the ability of artificial agents to mindread to two “mental states”, namely their encountering (i.e., a kind of simple perception) and their registration (i.e., a rudimentary form of believing). Some authors find this approach to be “a promising starting point to characterize mindreading abilities of artificial agents” (Strasser, 2020). However, this line of thinking seems in some sense to be similar to the proposal of new conceptual tools for forms of non-reciprocal or asymmetric sociality by Seibt (2014, 2017), which we already rejected due to moral and ethical considerations.

Another solution would be to provide an alternative to theory-theory and simulation theory as a basis for (mutual) understanding. This brings into play *interactionist* theories of mindreading as developed, among others, by Shaun Gallagher (2001, 2008, 2020). In contrast to theory-theory and simulation theory, Gallagher focuses on (mutual) understanding in embodied and enactive processes of interaction and assumes that mental states of others are not necessarily hidden away. Rather, they are accessible through the actions, bodily expressions, etc. of others. This is essentially based on two theses: first, that thinking is not necessary or essentially an internal matter; and second, that thinking is socially situated. As regards the former, Gallagher, by referring to Merleau-Ponty (1964), argues that at least a part of our thinking would take place through speech acts. Even more, thinking could be co-constituted by speech, gestures, and facial expressions. This would mean that which speech acts one performs, as well as their meaning, necessarily depends on the social context. However, this does not allude to the position that speech acts would only be social interventions. Rather, they are *inherently* social, insofar as speech acts can only be understood regarding their embeddedness in a certain social context.

From these two theses Gallagher concludes that mental states of others can be perceived *directly* in the context of social interaction. In fact, we can *see* the emotions and intentions of others if we were to understand them as patterns of situated physical movements and expressive properties in normative social contexts, and we can *hear* the beliefs of others when they communicate them to us. This would mean to hear the other thinking, since at least some of her beliefs are realized in the context of their linguistic expression in the first place.

Regarding our question of (mutual) understanding as necessary condition for human–robot joint interaction, Gallagher’s focus on embodied and enactive processes of interaction is of particular importance, as it shifts the question of necessary conditions for joint interaction towards the question of necessary conditions of such embodied and enactive processes. Consequently, if it was possible to interact with robots in embodied and enactive processes, it would also be possible to jointly interact with them. This again would set fairly high standards for the development of social robots, for then robots would have to be designed in a way that would render them embodied and enactive in the very same sense as humans are, according to Gallagher. Whether this can be accomplished is, however, an open question despite numerous research activities on this matter.

## Conclusion

At the center of our considerations was the question in what sense it could be legitimate to speak of human–robot *joint interaction* and in what sense we could speak of humans and robots sufficiently understanding each other to account for human–robot joint interaction. The discussion of modern approaches to the implementation of certain communicative properties of humans in robots turned out to be insufficient for enabling mutual understanding, if only because their reference to the human tendency to anthropomorphize merely establishes a *one-way relation*, in which certain human values, attitudes, characteristics, and abilities are projected onto robots.

The cybernetic transmission model of communication also was shown to be insufficient with a view to mutual understanding and joint interaction. Although it is, in fact, compatible with many technical approaches in social robotics, its inherent reduction of communication to mathematically quantifiable and predictable information processing and exchange does not seem to account for any plausible version of human communication, understanding and joint interaction. Following phenomenologists like Levinas, on the other hand, it could be stated that communication and understanding is not readily comparable to human communication and understanding, which would mean that a completely different approach is needed for human–robot joint interaction. However, it remained an open question how such an approach could be conceptualized. Finally, defining mutual understanding in terms of theory-theory, simulation theory and interactionism proved to be too rich in preconditions to be appropriately implemented in social robots.

To summarize, all approaches analyzed here are either implausibly reductionist or link demands to mutual understanding that make it questionable if artificial agents like social robots can satisfy them appropriately. Simultaneously, we face the fact that joint interaction with robots seems to be possible in certain contexts (Breazeal et al., 2016). In principle, two answers seem possible to this observation: first, it could be stated that human–robot communication, understanding, and joint interaction have to be understood in a less demanding sense than human–human communication, understanding and joint interaction, e.g., in accordance with a *Minimal Theory of Mind* (Butterfill & Apperly, 2013), or with a view to the proposal of non-reciprocal or asymmetric sociality (Seibt, 2014, 2017). This alternative, however, is to be rejected due to moral and ethical considerations. Second, at least if any of the discussed accounts of mutual understanding would be sufficiently plausible and would, thus, have to be adapted to robots to legitimately speak of human–robot joint interaction, it follows that human–robot communication, understanding, and joint interaction are, at least for the time being, not possible. Consequently, humans and robots may cause events in the world together, but joint interaction—in a more sophisticated understanding of the term—is only seemingly possible at the moment. It is then morally as well as ethically important to not raise expectations of human–robot communication, understanding, and joint interaction too high. It is not legitimate to speak of human–robot joint interaction in the sense we speak of human–human joint interaction. Hence, on a moral level it is not legitimate to have any moral expectations of robots. And it is not ethically justified to treat robots as moral subjects or moral patients.

## Footnotes

1 Joint action has to be distinguished from *collective action*, which refers “to any action performed by an entity which is a group of several individuals” and which *does not* “imply anything about actions taken by individual members of a collective” (Hammond, 2016).

2 For the purpose of this article, we leave aside the question whether this enables communication and *human* understanding of robots (referring to, e.g., emotions, reasons, and intentions) in a plausible sense, i.e., the question of what exactly humans would have to understand in robots.

3 Although, e.g., Weaver (1949) suspected that the aspect of meaning in principle could be included and various attempts have been made to do so (Bao et al., 2011).

4 In the following, we leave aside post-phenomenological approaches (cf., e.g., Ihde, 1990; Rosenberger and Verbeek, 2015) as they are mainly concerned with human-world relations mediated by technology and not so much with phenomena like communication, understanding and joint interaction as we discuss them here.
